# Editorial: Presenteeism in the Aftermath of COVID-19: New Trends and Contributions Regarding Sickness Presence at Work

**DOI:** 10.3389/fpsyg.2022.854976

**Published:** 2022-04-12

**Authors:** Aristides I. Ferreira, Merce Mach, Luis F. Martinez, Mariella Miraglia

**Affiliations:** ^1^Business Research Unit, Iscte – Instituto Universitário de Lisboa, Lisbon, Portugal; ^2^Faculty of Economics and Business, University of Barcelona, Barcelona, Spain; ^3^NOVA School of Business and Economics, Universidade NOVA de Lisboa, Lisbon, Portugal; ^4^Management School, Faculty of Humanities and Social Sciences, University of Liverpool, Liverpool, United Kingdom

**Keywords:** presenteeism, productivity, sickness, multilevel perspective, COVID-19, remote work

As Guest Editors for the Frontiers in Psychology (Organizational Psychology section), we are delighted to announce that 13 thought-provoking articles were accepted for inclusion in this Special Issue entitled “Presenteeism in the Aftermath of COVID-19: New Trends and Contributions Regarding Sickness Presence at Work”. We believe all these research projects make a substantial contribution to the literature and will set the tone for future avenues of research on sickness presenteeism.

In that sense, after a peer review that had the contribution of more than 30 high-quality international external reviewers with expertise in the domain of presenteeism, the 13 accepted papers highlight relevant contributions to the growing field of presenteeism. Undeniably, the COVID-19 pandemic experience has introduced conjunctural and structural challenges in society, economy, organizations, and individuals with determining impact in our life. More than ever, the act of going to work while ill (i.e., presenteeism) has been questioned and, consequently, the construct deserves to be studied in more detail. The therapeutical act of presenteeism (c.f., Karanika-Murray and Biron, 2020) will never be perceived the way as it was before. Going to work with a contagious disease like the flu, something normal in the past, and sometimes supported by companies with climates (Ferreira et al., 2019) and cultures (Simpson, 1998) of presenteeism, will not likely be observed or even reinforced in the same way. Therefore, this new pandemic imposed some challenges for employees and managers and, as usually, when unexpected events happen, science moves forward. These papers represent just a few examples of how something so relevant such as presenteeism found room for a new era of research. With this collection of articles, we seek to provide some guidance for both managers and employees by helping them to clarify the “should I stay or should I go to work while ill” *dilemma*.

In order to develop new research, more instruments are required in the field. This Special Issue includes two important psychometric contributions from Gelfand et al. and Shan et al. with new important scales to measure presenteeism. The scale developed by Shan et al. revealed good psychometric properties and is recommended for use in the health sector, particularly for nurses. Another validation study conducted by Gelfand et al. also provide a mixed methods design to provide psychometric evidence on the Valuation of Lost Productivity Questionnaire. This study was validated in a sample of 383 caregivers and provides evidence of feasibility and preliminary validity considering several outcomes, suggesting that this measure can be used as a tool to study presenteeism and absenteeism.

Other studies (e.g., Biron et al.; Chen et al.; Chou and Mach; Shimura et al.) adopted robust repeated-measures longitudinal methodologies to support their main findings and contributions to the field. For example, a six-months two-wave panel design study conducted by Chen et al. adopted the conservation of resources theory to explain a three-way interaction effect of presenteeism and social support at work, and collegial support on innovative behavior. This study is one of the first to link presenteeism and innovative behavior. Biron et al. provided a thought-provoking contribution by studying the impact of teleworking while sick in a three-way research design. Among other contributions to the workplace climate and working conditions, the authors showed that higher initial perceptions of psychological safety climate tend to reduce subsequent psychological demands which later decreases the decision to work while ill. The paper from Chou and Mach contributes to understand the psychological mechanisms influencing presenteeism over distinct timeframes (one week and 1 year), each leading to different personal and work outcomes. Among other notable contributions, the three-wave panel design provide evidence that presenteeism is positively associated with increased effort, work engagement, and job performance after 1 week. Then, the two-wave panel survey of before and after the first pandemic wave from Shimura et al. provide empirical evidence of the implications for people and organizations of the new scenario of working from home, as previous results had been conflicting on workers' mental health and presenteeism. Complementarily, the theoretical and conceptual study developed by Ferreira et al. provided a conceptual model where a new construct of remote-work presenteeism behavior mediated the relationship between different post pandemic health conditions (e.g., allergies, back pain, depression, anxiety) and future cumulative negative consequences.

Ruhle and Schmoll adopted a different methodological approach considering a qualitative study with a sample of 505 participants to explore (virtual) sickness presenteeism in the context of the COVID-19 pandemic. The authors addressed important questions, such as how virtual work affects the decision to disclose presenteeism behavior. Moreover, the authors provided relevant propositions to understand how the connection between telecommuting and virtual sickness presenteeism during a pandemic impact individuals and organizations.

Other important work presented in the Special Issue includes cross-sectional studies with big samples in sectors as distinct as education (e.g., Ferreira et al.), vocational counselors (Zürcher et al.), and self-employees (Vinberg et al.). For example, the work developed by Ferreira et al. addresses the role of productivity loss due to presenteeism in the relationship between observing cyberbullying situations among students and teacher burnout. Main findings show that teacher's productivity loss due to presenteeism mediated the relationship between observing cyberbullying incidents among their students and their burnout levels. Specifically, the effect of productivity loss due to presenteeism explained the effect of observing cyberbullying incidents on teachers' burnout levels. These results also shed light on the importance of wellbeing in the context of hostile situations. In the XXI century, with the rise of self-employees worldwide, Vinberg et al. developed an interesting study that may pave further research on the relationship between self-employees and presenteeism. In particular, the authors revealed that high workloads and increased demands on business operations (e.g., new product development, risk of bankruptcy and increased working hours) tend to explain the variance associated to sickness presenteeism among self-employed people. Another cross-sectional study of vocational counselors working from home during pandemic times (Zürcher et al.) illustrates how those professionals perceive themselves more productive and satisfied with their job, compared to working on-site.

As most studies on sickness presenteeism address both health and educational sector employees (Zhou et al., 2016), two studies included in this Frontiers Special Issue focus specifically on the IT professionals. Particularly, the cross-sectional manuscript written by Yang et al. covers the relationship between the event strength of co-worker presenteeism and innovative behavior among 374 IT professionals. The results showed the timing of co-worker presenteeism events moderated the relationship between the criticality of co-worker presenteeism events and promotion focus. Studying the same sector but including an experimental vignette study, Lohaus and Habermann focuses on the intricate decision-making process for or against working while ill. Drawing on expectancy theory, the authors found that the calculated and predicted decisions significantly matched the individuals' intentionally chosen decisions. The findings also provide insightful practical ideas for companies when managing interventions on attendance behavior.

Finally, these 13 papers included in this Frontiers in Psychology (Section: Organizational Psychology) Special Issue use robust and provocative theoretical approaches to provide evidence about the research developed around the construct of presenteeism in the specific context of the COVID-19 pandemic context. Most of these important contributions aim to reduce negative outcomes and to promote healthy organizational environments at different levels, so that companies, teams, and individuals could be more productive even in times of crisis. Moreover, their findings come from different theoretical backgrounds and cultural contexts, and the adoption of mixed and somewhat robust methodologies offer several clues for developing future studies and to implement multilevel interventions, particularly in the post-pandemic era.

We hope the scientific community can benefit from the theoretical and practical contributions derived from these contributions. Additionally, we expect that the main findings derived from these papers may attract the attention of the academicians in the field of presenteeism and other related-constructs (such as absenteeism) to inspire further research considering the aftermath of COVID-19.

## Author Contributions

All authors listed have made a substantial, direct, and intellectual contribution to the work and approved it for publication.

## Funding

This work was funded by Fundação para a Ciência e a Tecnologia (UID ECO/00124/2019, UIDB/00124/2020, UIDB/00315/2020, Social Sciences DataLab, PINFRA/22209/2016), POR Lisboa, and POR Norte (Social Sciences DataLab, PINFRA/22209/2016).

## Conflict of Interest

The authors declare that the research was conducted in the absence of any commercial or financial relationships that could be construed as a potential conflict of interest.

## Publisher's Note

All claims expressed in this article are solely those of the authors and do not necessarily represent those of their affiliated organizations, or those of the publisher, the editors and the reviewers. Any product that may be evaluated in this article, or claim that may be made by its manufacturer, is not guaranteed or endorsed by the publisher.
